# The effect of co-occurring lesions on leukaemogenesis and drug response in T-ALL and ETP-ALL

**DOI:** 10.1038/s41416-019-0647-7

**Published:** 2019-12-03

**Authors:** Paniz Tavakoli Shirazi, Laura N. Eadie, Susan L. Heatley, Timothy P. Hughes, David T. Yeung, Deborah L. White

**Affiliations:** 1 Cancer Program, Precision Medicine Theme, South Australian Health & Medical Research Institute (SAHMRI), Adelaide, SA Australia; 2 Faculty of Health and Medical Sciences, University of Adelaide, Adelaide, SA Australia; 3 Faculty of Sciences, University of Adelaide, Adelaide, SA Australia; 4 Australian Genomics Health Alliance (AGHA), Parkville, VIC Australia; 5 Royal Adelaide Hospital, Adelaide, SA Australia

**Keywords:** Cancer genomics, Acute lymphocytic leukaemia

## Abstract

Despite advances in the management of acute lymphoblastic leukaemia (ALL), current regimens fail to significantly transform outcomes for patients with high-risk subtypes. Advances in genomic analyses have identified novel lesions including mutations in genes that encode chromatin modifiers and those that influence cytokine and kinase signalling, rendering many of these alterations potentially targetable by tyrosine kinase and epigenetic inhibitors currently in clinical use. Although specific genomic lesions, gene expression patterns, and immunophenotypic profiles have been associated with specific clinical outcomes in some cancers, the application of precision medicine approaches based on these data has been slow. This approach is complicated by the reality that patients often harbour multiple mutations, and in many cases, the precise functional significance and interaction of these mutations in driving leukaemia and drug responsiveness/resistance remains unknown. Given that signalling pathways driving leukaemic pathogenesis could plausibly result from the co-existence of specific lesions and the resultant perturbation of protein interactions, the use of combined therapeutics that target multiple aberrant pathways, according to an individual’s mutational profile, might improve outcomes and lower a patient’s risk of relapse. Here we outline the genomic alterations that occur in T cell ALL (T-ALL) and early T cell precursor (ETP)-ALL and review studies highlighting the possible effects of co-occurring lesions on leukaemogenesis and drug response.

## Background

Acute lymphoblastic leukaemia (ALL) is a haematological malignancy of precursor B cell or T cell lineage that affects children and adults (85–75% and 15–25% of cases, respectively, for precursor B-ALL and T-ALL).^1–3^ T-ALL, which will be the focus of this article, arises due to infiltration of the thymus, lymph nodes, and bone marrow with T lymphoid precursors that harbour genetic and epigenetic mutations. Mutations in several key genes (e.g. *LMO2*, which encodes LIM domain only protein 2, and *LYL1*, which encodes lymphoblastic leukaemia derived sequence 1, both of which are key regulators of haematopoietic progenitor cell development) reprogramme T cell progenitors into pre-leukaemic stem cells (pre-LSCs).^4^ These mutated pre-LSCs can expand and acquire additional oncogenic mutations, resulting in a disease that differs greatly between individual patients.^5^

Therapy for T-ALL, a disease that is traditionally associated with inferior outcomes, is generally selected on the basis of a variety of factors, including age, white blood cell count, karyotypic alterations, and the immunophenotypic signature of leukaemic cells.^6,7^ These factors, in addition to treatment response (evaluation of minimal residual disease following induction therapy), are important indicators of prognosis and have facilitated risk stratification of patients.^1,6^ Optimised risk stratification, coupled with improved chemotherapy regimens and haematopoietic stem cell transplantation (SCT), has resulted in improved rates of survival, such that 80% of paediatric and 50% of adult T-ALL patients now achieve 5-year event-free survival.^7,8^ However, SCT, the only curative option in high-risk disease, might be limited by availability of matched stem cell/bone marrow donors and is associated with a substantial risk of SCT-associated morbidity and mortality.^9,10^ Current chemotherapy regimens are associated with both short- and long-term toxicity,^11^ such that even in survivors there might be long-term sequalae, including secondary malignancies.^12,13^ Furthermore, despite this improvement in rates of survival, relapsed or refractory T-ALL still occurs in approximately 20% of childhood cases and half of the adult cases, regardless of disease subtype (outlined below in the main text). Outcomes are extremely poor for relapsed disease^2,14^: in adult patients with subsequent relapse, the long-term survival rate remains <10%.^3^ In many cases, relapsed disease arises from the persistence of leukaemic T lymphoblasts in one or more sanctuary sites, such as the central nervous system (CNS).^15^ A recent study in precursor B-ALL has suggested that the presence of leukaemic cells with specific genetic determinants during disease development can increase the likelihood of relapse resulting from CNS disease,^16^ highlighting the need to correctly identify patients at risk of CNS involvement and potential relapse. Early identification is particularly important, as salvage for patients with refractory and/or relapsed disease, even with intensified chemotherapy regimens and CNS-directed treatment and/or SCT, might not improve the survival rate.^1,9,11^

Better risk stratification could improve outcomes in all cases, including in those with refractor/relapsed disease by identifying patients at risk of treatment failure who would benefit from increased monitoring and treatment intensification, as well as those who might safely de-escalate treatment. This might be achieved through a better understanding of disease biology and the genomic basis of T-ALL. Next-generation sequencing (NGS) has facilitated the detailed identification and characterisation of genomic alterations that are present in ALL, including single-nucleotide variants, copy number alterations, insertions, and deletions.^5,17,18^ Gene fusions, changes in gene expression,^18–20^ abnormal regulation of epigenetic-modifier genes, and epigenetic changes such as histone modification and DNA methylation have also been characterised.^21,22^ These alterations identified in T-ALL patients might provide alternative targets for improved treatment outcomes. However, the precise role of these alterations, and possible co-operation between mutations, in the induction of leukaemia, determination of drug response, and development of drug resistance remain to be defined and are the focus of this review.^23,24^

## Genomic profiling and genetic subtypes of T-ALL

**Fig. 1:**
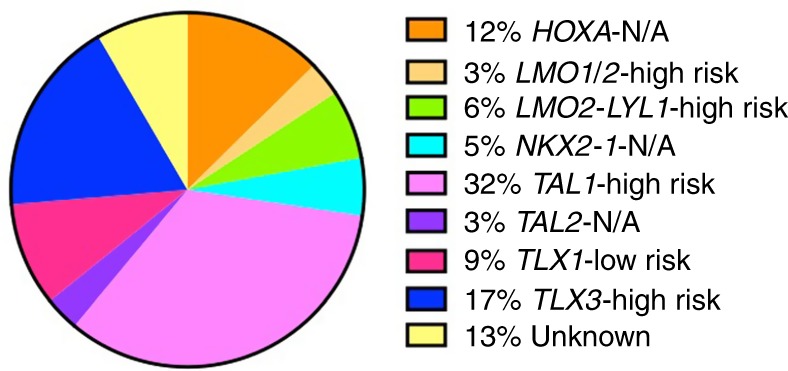
Key subgroups of paediatric and young adult T-ALL. Relative incidence and the prognosis of T-ALL subgroups. Compiled from refs. ^18,24,26^. N/A no data available.

Although the molecular classification of T-ALL is not as advanced as it is for other leukaemias such as precursor B-ALL, progress has been made in this endeavour over the past decade. Diverse subtypes of T-ALL have been identified based on unique immunophenotypes (Table 1), gene expression, and cytogenetics (Fig. 1).^25^ Depending on the maturation phase at which T cell development is arrested, T-ALL is subclassified into early T cell precursor (ETP)-ALL, a distinct subtype derived from the earliest cortical thymic cells; early T-ALL (pro- and pre-); late cortical T-ALL; and mature T-ALL (Table 1). These subtypes are distinguishable on the basis of their cluster of differentiation (CD) markers. In addition, different immunophenotypic subtypes have enrichment of particular gene alterations, which are discussed further in upcoming sections.

**Table 1:** Key CD antigen markers of T-ALL subtypes.

T cell subtypes	CD antigen markers
ETP	CD1a^–^, CD8^–^, CD4^–^, weak/focal CD3, weak or negative CD5, CD56^+^, aberrant expression of at least one myeloid marker (e.g. MAC1, GR1, KIT, CD13, CD33), and haematopoietic stem cell markers (e.g. CD34, CD25, CD44)
Pro-T	Cytoplasmic CD3^+^, surface CD3^–^, CD7^+^, CD5^–^, CD2^–^, CD1a^–^, CD34^+/–^, CD8^–^, CD4^–^
Pre-T	Cytoplasmic CD3^+^, surface CD3^–^, CD7^+^, CD5^+^, CD2^+^, CD1a^–^, CD34^+/–^, CD8^–^, CD4^–^
Cortical	Cytoplasmic CD3^+^, surface CD3^+/–^, CD7^+^, CD5^+/–^, CD2^+/–^, CD1a^+^, CD34^–^, CD8^+^, and CD4^+^
Mature	Cytoplasmic CD3^+^, surface CD3^+^, CD7^+^, CD5^+/–^, CD2^+^, CD1a^–^, CD34^–^, CD8^+^, or CD4^+^

This table was compiled based on information from refs. ^3,75,76,116^

A subset of T-ALL patients harbour chromosomal rearrangements that generally translocate homeobox (HOX) or transcription factor genes into positions adjacent to the T cell receptor locus (approximately 91% of cases).^18,26^ These chromosomal translocations result in altered expression of the transcription factors that subsequently lead to abnormal expression of genes involved in regulation of T cell development.^26^ Formerly, T-ALL was divided into four major groups, according to the distinct gene expression profile of these genomic translocations: *LYL1*, T cell leukaemia homeobox protein 1 (*TLX1;* initially named *HOX11*), *TLX3* (previously *HOX11L2*), and T cell ALL (*TAL1/LMO2*).^26^ However, a comprehensive genomic analysis of >260 paediatric and young adult T-ALL patients allowed further classification into eight major groups based on the translocated gene and its dysregulated expression: *TLX1*, *TLX3*, *TAL1*, *TAL2*, *LMO1/2*, *NKX2-1*, *HOXA*, and *LMO2-LYL1*.^18^ Furthermore, frequent translocation of T cell receptor genes to those encoding the basic helix-loop-helix transcription factor *OLIG2*, *MYC*, and the proto-oncogene *MYB* has been reported.^18^ The remaining 9% of cases harbour chimeric fusions involving miscellaneous genes such as *MLLT10*, *KMT2A* (previously *MLL*), *ABL1* (a non-receptor tyrosine kinase), and *JAK2* (encodes Janus kinase 2), which encode components of transcriptional regulation (*MLLT10*), epigenetic regulation (*KMT2A*), and kinase signalling pathways (*ABL1* and *JAK2*).^18,24^ Some of the more common chimeric fusions include *PICALM–MLLT10*, *MLLT10–KMT2A*, and *NUP214–ABL1*.^18^

Some of these recurrent lesions observed in T-ALL are similar to those observed in other haematopoietic disorders such as precursor B-ALL—for example, the *NUP214–ABL1* fusion.^27,28^ Tyrosine kinase inhibitors such as dasatinib and nilotinib are effective against *BCR–ABL1*-positive leukaemias and *ABL*-rearranged precursor B-ALL^27,29,30^ and show activity in vitro against *NUP214*–*ABL1* T-ALL; however, clinical data are limited in this setting and further investigation is warranted.^31–33^

**Fig. 2:**
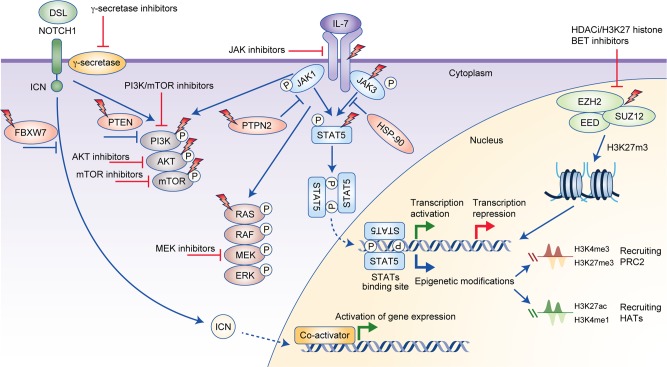
Overview of oncogenic pathways activated in T-ALL, the downstream signalling network of interleukin-7 (IL-7), NOTCH1, and polycomb repressor complex 2 (PRC2), and potential therapeutic targets. Binding of cytokine to the IL-7 receptor complex results in dimerisation of the receptor complex, which consequently phosphorylates JAK, as the cytokine receptor itself lacks intrinsic biological activity.^50^ Activated JAK1 and JAK3 induce phosphorylation of the STAT5 transcription factor, which, following dimerisation, translocates into the nucleus and stimulates gene expression.^50^ In addition, JAKs activate other downstream signalling cascades including PI3K–mTOR and RAS, which rationalises the use of combinations of inhibitors to promote cell death. STATs can also bind to the enhancer region of genes and modulate the epigenetic status of genes by depositing activating or repressive epigenetic marks through the direct recruitment of PRC2 members, histone acetyltransferases (HATs) or through regulation of their transcription.^114^ Abnormal NOTCH1 signalling can enhance IL-7R signalling.^41^ When the NOTCH1 receptor is activated in response to Delta-Serrate-Lag2 (DSL) ligand, signalling is then mediated by intracellular NOTCH1 (ICN), which functions as a transcription factor.^115^ Translocation of ICN to the nucleus and recruitment of co-activators subsequently activates downstream gene expression.^115^ Lightning bolts represent the proteins that are mutated in T-ALL. Red proteins are pathway regulators. Potential inhibitors of the proteins and pathways are indicated. BET Bromodomain and Extra-Terminal motif, HAT histone acetyltransferase, HDACi histone deacetylase inhibitor.

The genomic profile of lesions responsible for leukaemogenesis of T-ALL is further complicated by recurrent cytogenetic and molecular alterations that commonly occur in addition to the above-mentioned rearrangements. Frequently dysregulated pathways in T-ALL govern *NOTCH* signalling (60%), the JAK–signal transducer and activator of transcription (STAT) (25%) and phosphatidylinositol 3-kinase (PI3K)–mammalian target of rapamycin (mTOR) (29%) signalling pathways, RAS signalling (14%), and epigenetic regulation (68%)^18^ (Fig. 2). Interestingly, approximately 20% of T-ALL cases harbour three or more mutations in multiple signalling pathways that co-exist either in the same or separate leukaemic clones.^18^ The most common patterns of co-expression reported are an activating mutation in a component of the JAK–STAT signalling pathway coupled with secondary mutations to members of the JAK–STAT (~34%), RAS (~20%), or PI3K–mTOR (~10%) signalling pathways and a mutation to a member of the PI3K–mTOR signalling pathway with concomitant mutations involving PI3K–mTOR (~23%) and RAS (~7%) signalling.^18^ Mutations to these genes result in disruption of cellular processes such as cell proliferation, cell cycle control, T cell differentiation, and chromatin remodelling.^26^

### Lesions activating NOTCH1 signalling

The *NOTCH1* gene encodes a transmembrane receptor that is critical for determining T cell survival and fate specification. The NOTCH1 transmembrane receptor is necessary for directing pluripotent progenitors towards T cell fate and subsequent assembly of T cell receptor complexes.^34,35^ Inactivation of Notch1 in mice models demonstrated a blockage in T cell development and T cell lineage specification failure.^34^ Insertion and deletion mutations causing constitutive activation of NOTCH1 signalling are observed in >60% of T-ALL cases.^18,35^ Altered NOTCH1 signalling results in a massive expansion of immature T cells, increasing the risk of additional leukaemic lesion acquisition.^35–37^ Constitutive activation of NOTCH1 signalling can affect other signalling pathways including cell cycle and nuclear factor κ light-chain enhancer of activated B cells (NF-κB) signalling.^38^ Moreover, alterations in *NOTCH1* can also activate the PI3K–mTOR signalling pathway and increase *c-MYC* gene expression, which further promotes cell growth.^35,39,40^ Inactivating mutations in the gene that encodes the tumour-suppressor FBXW7, which regulates the proteasome-mediated degradation of NOTCH1, are also commonly observed in T-ALL patients and result in loss of NOTCH1 protein degradation and subsequent activation of NOTCH1 signalling.^18,35,41^

### Lesions that cause the loss of tumour suppressors and cell cycle regulators

Loss of cell cycle regulation and proliferation control are known to play a critical role in cancer development.^42^ Deletion of the cyclin-dependent kinase inhibitors *CDKN2A* and *CDKN2B*, which regulate the cell cycle, are observed in >70% of T-ALL cases.^18,43^ *CDKN2A* deletion frequently occurs with *CDKN2B* deletion as a result of instability of chromosome 9p21.3 in T-ALL.^18,44,45^ These genomic alterations are secondary events and co-operate with other initiating lesions (e.g. *NOTCH1* mutations) to facilitate development of T-ALL.^44,46,47^ Deletion of tumour-suppressor genes such as *CDKN2A/CDKN2B* promote development of T-ALL through loss of cell cycle control as well as failure to employ the p53 cell division check point and hinderance of apoptosis.^47^ Additional commonly mutated genes in this pathway are *CDKN1B* and *RB1*, which are also tumour suppressors and detected in approximately 15% of cases.^18^ Similarly, deletion of *CDKN1B* and *RB1* ultimately result in proliferation of T cells harbouring additional alterations to oncogenes and/or transcription factors that promote the onset of T-ALL.^47^

### Lesions activating the JAK–STAT pathway and receptor for interleukin-7 (IL-7R) signalling

Cytokine signalling through the IL-7R and resultant activation of the JAK–STAT pathway is essential in early lymphoid maturation and in the maintenance of B and T lymphocytes.^48,49^ The IL-7R complex predominantly signals through phosphorylation of JAK1 and JAK3 leading to activation of STAT5.^50^ In the T-ALL setting, recurrent activating mutations to *IL-7R* (~10%), *JAK1* (4%)*, JAK3* (16%), *SH2B3* (encodes an adaptor protein; 1%), and *STAT5B* (4.5%) result in constitutive JAK–STAT signalling.^45,51–53^ Co-expression of mutations in these genes is repeatedly observed, with approximately 6% of T-ALL patients harbouring a combination of mutations.^45^ In addition, alterations in either *JAK2* or *TYK2*, which encodes another member of the JAK family, have also been reported in T-ALL, although they are uncommon.^19,54,55^ Although, no underlying lesion of *CRLF2* (encodes cytokine receptor like factor 2) has been identified in T-ALL, overexpression of this gene has been noted in some cases resulting in activation of JAK–STAT signalling through the thymic stromal lymphopoietin receptor complex.^56^ In addition, *CRLF2* overexpression is associated with an inferior event-free survival rate.^56^

### Lesions activating RAS and PI3K–mTOR pathway

RAS proteins are small GTPases responsible for transmitting activation signals from cytokine receptors, cell surface receptors such as the T cell receptor, receptor tyrosine kinases, or other downstream signalling pathways, including PI3K–mTOR.^57^ The most commonly mutated genes in the RAS pathway include *NRAS* (~8%), *NF1* (~4%), and *KRAS* (~2%), and the mutations frequently confer increased activation.^18,45,58^

PI3K–mTOR signalling allows the proper development of T cells and promotes the survival of developing T cells.^59^ PI3K pathway mutations are more prevalent in *TAL1* cases in a T-ALL setting.^18^ Constitutive activation of the PI3K–mTOR pathway is commonly associated with inhibitory mutations to *PTEN* (~20%), *PIK3R1* (~6%), and *AKT1* (~2%) as well as deletion of the phosphatase *PTPN2* (~5%).^18,60–62^

### Lesions in epigenetic regulators

Epigenetic modifications, including histone modification, DNA methylation, and nucleosome remodelling, enable activation or repression of gene expression.^63^ The prevalence of lesions in epigenetic regulators in T-ALL suggests an important role for epigenetic regulation in disease development. Mutations in epigenetic modifiers can alter the accessibility of certain parts of chromatin to transcription factors, and if this process occurs at the incorrect stages of T cell maturation, aberrant gene expression can occur, contributing to disease pathogenesis.^64^ Genes encoding chromatin modifiers and epigenetic regulators that are recurrently mutated in T-ALL have a higher incidence among *TLX3-*positive and *TLX1*-positive cases—in particular, inactivating mutations to the gene encoding the plant homeodomain-like finger family member *PHF6*, which occur in approximately 16% of paediatric and 33% of adult T-ALL cases.^65^ Loss-of-function mutations in the genes encoding the core components of the polycomb repressor complex 2 (PRC2)—*EZH2*, *SUZ12*, and *EED*—are present in 25% of T-ALL and 42% of ETP-ALL cases.^58,66^ In addition, changes in EZH2 expression levels and alterations in genes that encode PRC2-associated proteins, such as the methyltransferase *DNMT3A* and the transcriptional repressor *JARID2*, have been identified in T-ALL.^67^ Recurrent deletions and nonsense and frameshift mutations (mainly positioned in the SET domain of *EZH2* and the zinc finger VEFS-Box of *SUZ12*) predicted to result in a truncated form of EZH2 and SUZ12 have been reported, suggesting that the PRC2 complex normally confers a tumour-suppressor role against T-ALL.^58,66,67^ PRC2 plays an important role in initiating/maintaining di- and tri-methylation of lysine 27 on histone 3 (H3K27me2/3),^68^ which functions as a chromatin-repressive mark and is thus associated with gene silencing. Therefore, loss-of-function mutations to PRC2 complex members can consequently prevent silencing of specific oncogenes confirming the role of the PRC2 complex as a tumour suppressor.^66^ However, upregulation of *EZH2* has been reported in other cancers such as breast and prostate, and increased expression of *EZH2* promoted cell proliferation in some prostate cancer cell models.^69,70^ Furthermore, gain-of-function mutations (such as tyrosine 641 positioned in the SET domain of *EZH2*) in B cell lymphoma increase the enzyme’s catalytic activity and affinity for H3K27me3, suggesting an oncogenic role for this complex in these settings.^71,72^ By contrast, loss-of-function mutations and deletions in *EZH2* and *SUZ12* are associated with myeloid leukaemia and myeloproliferative neoplasms.^73,74^ It is evident that the function of the proteins might be altered depending on the regions in which the mutation occurs.

Some of the above-mentioned mutations are enriched in particular genomic subgroups and/or T cell developmental stages. Often mutations co-occur with other alterations or are common across all subgroups. These differences in mutation occurrence and co-occurrence are further discussed in this review.

## Early T cell precursor ALL

ETP-ALL is a T-ALL subtype characterised by the abnormal expression of stem cell or early progenitor and myeloid markers and the reduced or absent expression of common T cell markers, including CD4, CD8, CD1a, and CD5 (Table 1).^75^ This immunophenotype signature distinguishes ETP-ALL from all other T-ALL subtypes, including early T-ALL (pro- and pre-), late cortical T-ALL, and mature T-ALL (non-ETP-ALL). Several early clinical studies have suggested that ETP-ALL is a high-risk disease, with higher levels of measurable residual disease, chemoresistance, and relapse compared with non-ETP-ALL cases.^75–78^ However, this is not without conjecture, and studies using a risk-adapted approach with intensified initial treatment demonstrated that the rates of 5-year event-free and overall survival do not differ significantly between ETP-ALL and non-ETP-ALL.^79,80^

There is no unifying genetic lesion that occurs in ETP-ALL, although there is a suggestion that ETP-ALL have distinct mutation patterns compared with non-ETP-ALL. The genomic alterations present in ETP-ALL are enriched for mutations in transcriptional, epigenetic, and signalling genes that are more characteristic of the gene expression and mutational profiles observed in myeloid malignancies such as acute myeloid leukaemia, as expected by their immunophenotype.^18,75,81^ NGS has demonstrated vast genomic heterogeneity^18,58^ with multiple, recurrently mutated pathways comprising deletions, translocations, and sequence mutations in genes responsible for transcriptional regulation, noted in approximately 89% of ETP-ALL cases, including *BCL11B* (encodes BAF chromatin remodelling complex subunit BCL11B), *ETV6* (encodes ETS variant 6), *RUNX1* (encodes runt-related transcription factor 1), and *WT1* (encodes Wilms tumour protein 1), activation of JAK–STAT and IL-7R signalling (approximately 47% of ETP-ALL cases, including *JAK1*, *JAK3*, *IL7R*, and *SH2B3*), activation of RAS signalling pathways (approximately 36% of ETP-ALL cases, including *NRAS*, *KRAS*, and *PTPN11*), and histone modification (approximately 84% of ETP-ALL cases, including *PHF6*, transcriptional repressor *CTCF*, *EZH2*, and *SUZ12)*.

While enrichment of mutations in genes related to epigenetic regulation, JAK–STAT, and RAS signalling occurs in ETP-ALL in comparison with other T-ALLs, alterations in genes associated with cell cycle arrest (e.g. *CDKN2A/CDKN2B*) are less common.^18^ A higher prevalence of ETP-ALL cases occurs in the *LMO2/LYL1* and *TLX3*-mutated subgroups,^18^ and ETP-ALL patients demonstrate a similar mutational profile to that of acute myeloid leukaemia (AML) patients, with a haematopoietic stem cell-like gene expression profile.^77,82^ This suggests that ETP-ALL might arise in very early progenitor cells with multi-lineage potential. Characteristic gene alterations involve *FLT3*, including mutations in the tyrosine kinase domain and internal tandem duplications (noted in 26% of ETP-ALL case), commonly detected in AML,^18,82^ *IL-7R* mutations (10%),^18^ and mutations in the PRC2 core components *EZH2* (15%), *SUZ12* (15%), and *EED* (10%).^18^ ETP-ALL patients harbouring mutations in PRC2 core components have poor outcomes, particularly in *EZH2*-mutated cases, with an estimated 60% of these patients relapsing within 5 years.^58^ Novel targeted treatment approaches are required based on an individual’s mutational profile in order to improve survival outcomes in patients with ETP-ALL.

## Co-occurrence of genomic events: random or systematic?

As discussed, T-ALL is a genomically heterogeneous and complex disease, with individual patients harbouring a diverse range of multiple protein-altering lesions.^17,18,58^ A comprehensive genome-wide mutational profile of a large cohort of T-ALL patients suggested that multiple mutations that affect signalling pathways and epigenetic regulation and transcription and ultimately clinical outcome repeatedly co-occur within specific subgroups (Supplementary Table S1).^18^

Mutated genes that frequently co-occur within individuals include *PHF6* and *DNM2* (encodes dynamin 2), *RB1* and *DLEU1* (a long non-coding RNA) or *BCL11B*, and *MLLT10* and *ETV6* (Supplementary Table S1).^18^ In addition, associations have also been observed between *TCF7* (encodes transcription factor 7) and *JAK3* or *CDKN1B*, *ETV6* and *CDKN1B*, *NRAS* and *WT1*, and *LEF1* (encodes lymphoid enhancer-binding factor 1) and *PTEN* (Supplementary Table S1).^18^ *PTEN* mutations were detected mainly in patients within the *TAL1* and *LMO2* subgroups.^83^ Interestingly, *TAL1*-rearranged xenograft models acquired sub-clonal *PTEN* microdeletions during leukaemia development that were not present in the patient cells from which the xenografts were derived.^84^ This suggests that the initiating lesions might drive the acquisition of additional mutations in preferred partner genes and reinforces the hypothesis that the abundance of *PTEN* mutations in *TAL1* and *LMO2* subgroups is not random.

As both *NOTCH1* activation and *PTEN* deletion constitutively activate the PI3K–mTOR signalling pathway,^35,62^ it is likely that either alteration is sufficient for pathway activation; indeed, studies suggest that most *PTEN/AKT*-mutated cases lack *NOTCH1*-activating mutations.^83^ Conversely, in cases where there is *PTEN* deletion as well as activation of *NOTCH1*, in vitro resistance to *NOTCH1* inhibitors can occur, highlighting the need for new therapeutic strategies based on co-occurring genomic alterations.^60^ In addition, mice with mutant *TLX1*-induced leukaemia recurrently develop *Bcl11b* mutations, suggesting a co-operative function between these two alterations.^85^ Another study highlights the significant occurrence of mutations in the X-linked driver genes *USP9X* and *MED12* with mutations in *PHF6* in a paediatric T-ALL cohort.^17^ A number of studies observed enrichment of mutations in *IL-7R–JAK* signalling components in patients with ETP-ALL as well as those in the *TLX3* and *HOXA* subgroups.^45,52^ However, another study suggested the frequency of *IL-7R* mutations was similar across all disease groups.^18^ Furthermore, a significant association of *IL-7R* and *JAK3* gene mutations with mutations in *PHF6*, *WT1*, and PRC2 components has been observed.^45^

Targeted sequencing of T-ALL patient samples has also demonstrated the frequent co-occurrence of PRC2 component mutations with *NOTCH1*-activating mutations.^66^ The same study also suggested that silencing of the PRC2 components *EZH2* and *SUZ12* upregulates typical NOTCH1 target genes including *HES1* and *DTX1* and results in decreased apoptosis in the presence of a γ-secretase inhibitor that inhibits NOTCH1 signalling.^66^ This suggests that the loss of PRC2 potentiates NOTCH1 protein signalling and increases NOTCH1 target gene expression, despite inhibition of NOTCH1. Chromatin immunoprecipitation sequencing (ChIP-seq) revealed a notable loss of H3K27me3 on NOTCH1 target genes that overlapped with regions of NOTCH1 binding within the target gene transcriptional start sites.^66^ This implies that NOTCH1 signalling is normally associated with reduced PRC2 complex activity.^66^ These observations further support a functional association between the PRC2 complex and NOTCH1 signalling, highlighting that co-operation between PRC2 loss and *NOTCH1*-activating mutations can lead to enhanced tumorigenic potential in T-ALL cases.

The presence of multiple lesions in leukaemic T cells implies a potential functional connection between mutations during the development of leukaemia and progression of the disease. The fact that different mutations co-occur at different stages of T cell development also suggests a functional connection. It is plausible that the accumulation of lesions within a patient is not a random event and that acquisition of specific combinations of alterations during the progression of leukaemia might contribute to disease pathogenesis. However, the functional patterns of co-occurrence, the exact mechanisms, the potential of synthetic lethality, and prognostic implication have not been fully elucidated and require further investigation.

## Co-operation of different genomic lesions and increased susceptibility for distinct leukaemic profiles

Some mutations, alone or in combination with other specific lesions, can result in developmental arrest at different stages of T cell development to promote leukaemogenesis.^86–88^

### IL-7R-activating mutations alone and in association with other lesions

Activating mutations in mouse *Il-7r*, which are homologous to two *IL-7R* mutations identified in human ETP-ALL (*IL7R-241-242TC* and *IL7R-GCinsL243*),^58^ result in an aggressive ETP-like disease in mice.^86^ Transduced thymocytes expressing these mutations stop maturing at the double negative 2 (DN2, CD4^–^CD8^–^) stage of T cell differentiation ex vivo compared with thymocytes transduced with wild-type *Il-7r* and/or empty vector. In addition, development of leukaemia with a distinct immature phenotype (aberrant expression of CD44, myeloid markers, and intracellular CD3), similar to that observed in ETP-ALL patients, was detected in mice transplanted with *Il-7r*-mutated thymocytes.^86^

Another recent study investigated the functional association of *IL-7R* with *NRAS*, *NUP98–HOXD13* alterations, and TLX3 overexpression in mice. In contrast with previous studies, *Il-7r*-mutated mice developed multisystemic inflammatory disease rather than leukaemia.^89^ However, these opposing observations could be the result of different experimental models. The same non-leukaemic disease phenotype was also observed in mice that received cells expressing both *TLX3* and *Il-7r* mutations,^89^ suggesting that overexpression of TLX3 and mutations to the *Il-7r* gene are not sufficient for the development of T-ALL. However, mice transplanted with cells expressing both *Il-7r* and *NRAS* mutations developed fully penetrative polyclonal T-ALL with an immunophenotype more characteristic of mature disease, including the expression of CD4, CD8, CD3, CD90.2, and TCRβ.^89^ Furthermore, mice transplanted with thymocytes transduced with *Il-7r* and *NUP98–HOXD13* developed AML.^89^ Taken together, results from this study suggest that the polyclonal nature of the leukaemic cells might be important for disease development and that different combinations of mutations can result in different disease subtypes.

### Ezh2 inactivation with activating NRAS mutations

Deletion of the *Ezh2* gene in mice resulted in an immunophenotypically heterogeneous leukaemia with differing expression levels of CD4 and CD8. However, the majority of leukaemic cells were CD4^–^CD8^–^, and all leukaemic cells were CD3^+^.^67^ This is consistent with an early T-ALL immunophenotype, highlighting a premature block in T cell development. Another study used a mouse model to highlight the co-operation of deletion of the PRC2 components *Ezh2* and *Eed* with the *NRAS* Q61K mutation on a *Cdkn2a*^*−/−*^ background. In this second study, an aggressive leukaemia developed with shortened latency in comparison with *NRAS*-mutated models with intact PRC2 function.^88^ Furthermore, *Ezh2* inactivation generated an ETP-like immunophenotype (CD4^–^/CD8^–^/CD5^–^/Mac1^+^/GR1^+^) when combined with an *NRAS-*driven leukaemia.^88^ This is in stark contrast to the immunophenotype of disease with *NRAS* mutations alone (CD4^+^/CD8^+^/CD25^–^/CD44^–^), suggesting a less stringent differentiation block at the double-positive stage of T cell development.^88^ Subsequent investigation of the gene expression profile of the leukaemic cells suggested that inactivation of *Ezh2* resulted in enrichment of genes that are highly expressed in ETP-ALL.^88^ In addition, inactivation of *Ezh2* promoted the expression of genes that are normally epigenetically silenced via the H3K27me3 mark in mature T cells.^88^ Interestingly, increased phosphorylation of STAT3 and increased mRNA expression of *Il6ra* was noted in *Ezh2*-knockout cells, implying a co-operative mechanism between JAK–STAT pathway and PRC2 members.^88^

### Inactivation of Ezh2 and Runx1

A 2018 study investigated the possible co-operation of *Ezh2* with *Runx1* in dual knockout mice compared with individual knockouts and wild type.^87^ While all three knockout models (both individual knockouts and the double knockout) developed leukopenia, only dual inactivation of both *Ezh2* and *Runx1* resulted in a significant increase in the number of immature T cells (CD4^–^/CD8^–^/CD44^+^/CD25^–^/Kit^+^/Flt3^+^) and DN2 cells (CD4^–^/CD8^–^/CD44^+^/CD25^+^). Inactivation of *Ezh2* alone resulted in increased numbers of DN3 cells (CD4^–^/CD8^–^/CD44^–^/CD25^+^).^87^ Interestingly, addition of a Ras signalling pathway mutation (*FLT3*–*ITD*) to *Ezh2*–*Runx1* dual knockout mice resulted in the development of a highly aggressive ALL, with an increase in the number of immature T cells. Conversely, models with a *FLT3*–*ITD* mutation but functional *Ezh2* and *Runx1* resulted in a reduction in the number of immature T cells.^87^ Of clinical importance, *Ezh2*–*Runx1* dual knockout plus *FLT3*–*ITD* models have demonstrated constitutively activated RAS signalling in comparison with marginal activation in either dual knockout or *FLT3*–*ITD* mice.^87^

### Activating mutations in HOXA9 and JAK3

Another study by de Bock et al.^90^ indicates the co-operation of *HOXA9*- and *JAK3*-activating mutations in the development of leukaemia. In a transgenic mouse model, the co-expression of *HOXA9*- and *JAK3*-activating mutations resulted in a polyclonal leukaemic immunophenotype with cells exhibiting lymphoid (CD8^+^) or myeloid (CD4^–^, CD8^–^, CD16/32^+^, CD11b^+^) expression patterns.^90^ Increased activation of JAK3–STAT5 was noted in *HOXA9*–*JAK3* double mutant cells (both ex vivo and in vivo) in comparison with cells in which only *JAK3* was mutated. Interestingly, ChIP-seq analysis revealed that HOXA9 and STAT5 co-occupy similar genomic regions; when linked with gene enrichment data, this suggests that HOXA9 can promote the transcription of STAT5 in the absence of cytokine-mediated JAK3 activation.^90^ In addition, RNA expression data suggested that cells with dual *HOX9A*–*JAK3* mutations underwent differential clustering from cells with either *JAK3* or *HOXA9* mutations and indicated enhanced expression of the STAT5 target gene PIM1, a serine/threonine kinase that regulates several oncogenic processes, in *HOX9A*–*JAK3* double mutant cells.^90^ Importantly, the co-occurrence of *JAK3* and *HOXA* mutations has been acknowledged as being statistically significant in paediatric/young adult patients with T-ALL (Supplementary Table S1).^18,90^

### Activating lesions involving TLX1 and ABL1

*NUP214*–*ABL1* fusions are almost exclusively reported in *TLX3-*positive and *TLX1*-positive T-ALL cases (Supplementary Table S1).^18,61^ A study by Vanden Bempt et al.^91^ demonstrated the functional significance of constitutive activation of the ABL kinase (following fusion of *ABL1* to *NUP214*) and TLX overexpression in driving T-ALL. Interestingly, expression of NUP214–ABL protein alone did not induce leukaemic development, suggesting that, in contrast to other oncogenic ABL fusion proteins (e.g. BCR–ABL), NUP214–ABL in isolation is an inadequate oncoprotein.^91^ However, co-expression of *NUP214*–*ABL1* and *TLX1* in a mouse model resulted in development of an aggressive cortical-like T-ALL (CD4^+^/CD8^+^/CD3^+^) with a shorter latency compared with leukaemia resulting from *TLX1* alterations (long latency).^91^ RNA-seq analyses demonstrated enhanced JAK–STAT activation through increased STAT5 activation, as well as activation of downstream target genes (including *Myc* and *Bcl-2*), in *NUP214*–*ABL1*–*TLX1* double mutant mice compared with single mutant and/or wild-type mice.^91^ Interestingly, subsequent investigation revealed enhanced chromatin accessibility for differentially expressed STAT5 target genes in *NUP214*–*ABL1*–*TLX1* double mutant cells, as determined by H3K27 acetylation.^91^ Furthermore, Chip-seq analysis indicated co-binding of STAT5, TLX1, BRD4 (a member of bromodomain and extra-terminal motif [BET] family proteins), and MYC in the enhancer regions of STAT5 target genes (including *Myc* and *Bcl-2*). These findings highlight the co-operative regulatory effect of MYC and BCL-2 with STAT5 and TLX1 in *NUP214*–*ABL*–*TLX1*-induced leukaemia^91^ and suggest dual targeting with BET and/or BCL-2 inhibitors may be beneficial in this setting (discussed further below).

## Therapeutic possibilities

From a clinical standpoint, targeting known, recurrent combinations of mutations with appropriate inhibitors has been the focus of a number of preclinical investigations.

### JAK inhibitors alone and in combination

In a study by Treanor et al.,^86^ the JAK inhibitor ruxolitinib prolonged the survival of mice with ETP-ALL resulting from *Il-7r* mutations but failed to induce a significant anti-leukaemic response. Another study in mice harbouring both *NRAS* and *IL-7R* mutations investigated the effect of RAS and JAK–STAT signalling pathway inhibition using trametinib (a mitogen-activated protein kinase kinase [MEK] inhibitor) and ruxolitinib. Results demonstrated a significant decrease in leukaemic progression and infiltration compared with single-agent treatment cohorts.^89^ A third study found significantly increased signalling through the JAK–STAT pathway arising from activating mutations in *NRAS* in conjunction with *Ezh2* deletion.^88^ This contrasted with the marginal activation of JAK–STAT signalling observed when *NRAS* was mutated in isolation, justifying the use of ruxolitinib to target leukaemic cells with this combination of mutations.^88^ However, ruxolitinib demonstrated less potency against cells expressing mutated *NRAS* in combination with *Ezh2* deletion in vitro compared with cells expressing mutated *NRAS* alone.^88^ Taken together, these results suggest that patients with mutations to PRC2 components (such as *EZH2*), as well as unexplained JAK–STAT pathway activation, would benefit from a combination of epigenetic or MEK inhibition and possible JAK–STAT inhibition, dependent on the secondary alterations present.

In 5/6 ETP-ALL xenografted mice with JAK–STAT pathway overactivation, monotherapy with ruxolitinib significantly decreased the number of peripheral and splenic blast counts.^92^ The remaining xenograft did not have increased JAK–STAT activation and was less responsive to ruxolitinib, with only a decrease in splenic blast count. Interestingly, this xenograft had a mutation in *PTPN11*, with consequent activation of RAS signalling, and thus might have benefitted from the addition of MEK inhibitors.

Inhibition of JAK3 with tofacitinib (approved by the Food and Drug Administration [FDA] for the treatment of rheumatoid arthritis) in *HOX9A*–*JAK3-*mutated mice resulted in a reduction in spleen weight and in white cell count after 20 days.^90^ Given the increased expression of PIM1 in *HOX9A*–*JAK3*-mutated mice, the use of a PIM1 inhibitor in combination with inhibition of JAK–STAT might be of value. This combination has been tested but in xenograft models harbouring only JAK3 mutations. Treating these mice with AZD1208 (a PIM1 inhibitor) resulted in an increased attenuation of *JAK3*-mutated leukaemia compared with that seen following ruxolitinib treatment. Understandably, the best response was achieved using both inhibitors in combination.^90^ However, AZD1208 is not currently FDA approved and further development of this drug has been terminated owing to a lack of antitumour effects in AML.^93^ However, other PIM inhibitors, such as LGH447, are currently undergoing clinical trials. The use of JAK and PIM1 inhibitors in combination might warrant further study as increased levels of PIM1 were also reported in T-ALL patients harbouring *IL7R*, *JAK3*, and *JAK1* mutations, not only in *HOXA*-mutated cases.^90,94^

### BET inhibitors

In a study by Booth et al.^87^ investigating the co-operation of *Ezh2*, *Runx1*, and *FLT3* alterations in T-ALL, BET inhibitors effectively inhibited leukaemic cell growth in vitro and reduced tumour burden in *Ezh2*^*–/–*^*Runx1*^*–/–*^ *Flt3*–*ITD* mice. In this mouse model, PRC2 complex inactivation occurs resulting in the loss of the repressive H3K27me3 mark leading to subsequent increases in H3K27 acetylation.^87^ This epigenetic switch has been observed in previous studies and triggers the recruitment of BET proteins to the acetylated lysine sites.^95,96^ BET inhibitors are a class of epigenetic inhibitors that bind to members of the BET family of proteins (BRD2, BRD3, BRD4), preventing these proteins from binding to transcription factors and acetylated histones and are currently undergoing clinical trials.^97^ These data support the use of BET inhibitors in leukaemia in which there is inactivation of a component of the PRC2 complex. Indeed, combined ChIP-seq and RNA-seq analyses demonstrated increased H3K27 acetylation for differentially expressed genes in an *Ezh2*–*Runx1* dual knockout with *FLT3*–*ITD* mouse model compared with wild-type counterparts.^87^ Nevertheless, this disease profile might also benefit from RAS and/or JAK–STAT pathway inhibition, in addition to BET inhibition, as the gene expression analysis revealed an upregulation of the genes involved in activation of these pathways. However, these speculations require confirmation in an experimental model.

### BCL-2 family inhibition

A xenograft study investigating the effect of the BCL-2 inhibitor venetoclax, which has clinical efficacy in other settings, demonstrated the poor efficacy of this drug on T-ALL and ETP-ALL xenografts, despite the fact that high BCL-2 expression was positively correlated with a response in *KMT2A* (*MLL*)-rearranged and precursor B-ALL xenografts.^98^ This is most likely the result of high levels of BCL-X_L_, which is not targeted by venetoclax, indicating the need to concurrently inhibit this pro-survival protein. Although it is evident that patients can benefit from the addition of inhibitors to their chemotherapy regimen, precision therapeutics requires careful investigation of each patient’s genomic profile to ensure the best clinical outcome.

### Combined inhibition of JAK–STAT pathway with ABL, BET, and/or BCL-2 inhibitors

The use of BCL-2 and BET inhibitors in combination with ABL inhibitors might be beneficial. Treatment of xenograft-derived cells harbouring both *NUP214–ABL1* and *TLX1* alterations with imatinib (ABL kinase inhibitor) and JQ1 (BET inhibitor) or venetoclax, resulted in significantly reduced viability compared with treatment with each agent individually.^91^ The association of *NUP214*–*ABL1* fusion with *TLX1* alterations is further confirmed by the fact that patients with *NUP214*–*ABL1-*positive T-ALL consistently demonstrate upregulation of *MYC* and *BCL-2*.^18^ This observation might explain the variable clinical success of ABL kinase inhibitors against *NUP214*–*ABL1-*positive T-ALL^32,33,99^ and reinforces the benefits of combinational therapy with BCL2 and/or BET inhibitors for these patients.

### Immunotherapy

Immune-based therapeutic agents (including monoclonal antibody, chimeric antigen receptor-T cells and bispecific T cell-engaging antibodies) are undergoing preclinical studies^100–102^ and/or clinical trials (e.g. NCT03384654 and NCT03860844) against T-ALL. Unlike precursor B cell ALL, the use of immunotherapies against T-ALL in the clinic has been limited due to lack of unique targets that discriminate between leukaemic and healthy T cells and target-driven fratricide.^103^ It is plausible that a patient’s genomic background could influence response to immunotherapy through different combinations of mutations resulting in differential expression of CD markers. For instance, activating *IL7-R* and *JAK1* mutations were reported in combination with loss-of-function mutations and subsequent downregulation of CD45 in some T-ALL cases.^104^ In the precursor B cell ALL setting, differential expression of CD markers has been reported with enriched expression of CD27 observed in patients harbouring *ETV6*–*RUNX1*, *BCR*–*ABL1*, and *CRLF2* rearrangements.^105^ However, while no equivalent associations have been reported in the T-ALL setting, an investigation of the potential relationship between co-occurring mutations and response to immunotherapy is warranted but is not part of this review.

Collectively, it is possible that additional co-operating lesions are responsible for driving ETP-ALL or different T-ALL phenotypes. This accumulation of lesions can lead to the activation and/or inactivation of specific sets of oncogenes and tumour-suppressor genes, respectively, raising the possibility that the therapeutic outcome of these patients could be improved by adding targeted inhibitors to their therapy regimen. This article also highlights that the identification, and subsequent targeting, of driver pathways might be critical in some cases for an effective therapy outcome and prevention of disease relapse. Prior to the clinical adoption of this strategy, however, further research, including the screening of multiple drug candidates and their synergistic effect, is necessary to inform the best therapeutic combination required for specific sets of alterations—a key step that has been absent from previous studies.

## Future challenges

Important advances have been made in understanding the biology underlying T-ALL and determining the genomic lesions associated with this disease. However, substantial gaps in our knowledge remain. To date, most studies have investigated single lesions to validate transforming potential and drug responsiveness and have used transduced cell lines or murine models.^54,86,106,107^ Although these are well-established approaches, from a clinical point of view, each patient has an average of 10–20 genomic lesions.^45,108,109^ It is entirely plausible that co-operation of multiple mutations might boost the activation of specific signalling pathways; however, the significance of multiple lesions in combination has been poorly investigated and its importance might be underestimated.

Comprehensive studies using diagnosis and relapse patient material and xenograft models have demonstrated that the primary mechanism of relapse is the selection for pre-existing sub-clones and additional mutations detected in pre-leukaemic ancestral cells or primary leukaemia.^110–113^ Understanding the mutational patterns and the probability of each sub-clone’s ability to predominate, and their co-operative effect with driver mutations, might lead to improved clinical triaging of patients with different subtypes of T-ALL. However, this approach will require extensive modelling of different combinations of mutations, comprehensive RNA and protein profiling, and high-throughput drug screening. In the era of precision medicine and targeted therapy, it is important to understand the functional significance of co-operative events in order to use the most effective combination of inhibitors, at the right time in disease evolution, for each patient. The ultimate goal is to provide a highly efficient precision therapy approach that is tailored to the individual, with the lowest toxicity, to increase long-term, relapse-free survival rates in T-ALL.

## Supplementary information

Supplementary Table S1. Summary of genomic lesions associated with specific subgroups of T-ALL and potential co-existing genomic alterations

## Data Availability

Not applicable.
